# Can Coronaviruses Steal Genes from the Host as Evidenced in Western European Hedgehogs by EriCoV Genetic Characterization?

**DOI:** 10.3390/v12121471

**Published:** 2020-12-20

**Authors:** Luca De Sabato, Ilaria Di Bartolo, Maria Alessandra De Marco, Ana Moreno, Davide Lelli, Claudia Cotti, Mauro Delogu, Gabriele Vaccari

**Affiliations:** 1 Department of Food Safety, Nutrition and Veterinary Public Health, Istituto Superiore di Sanità, 00161 Rome, Italy; luca.desabato@iss.it (L.D.S.); ilaria.dibartolo@iss.it (I.D.B.); gabriele.vaccari@iss.it (G.V.); 2 ISPRA Institute for Environmental Protection and Research, 40064 Ozzano dell’Emilia, Italy; 3 Virology Unit, Istituto Zooprofilattico Sperimentale della Lombardia e dell’Emilia-Romagna, 25124 Brescia, Italy; davide.lelli@izsler.it; 4 Department of Veterinary Medical Sciences, University of Bologna, 40064 Ozzano dell’Emilia, Italy; claudia.cotti@unibo.it (C.C.); mauro.delogu@unibo.it (M.D.)

**Keywords:** *Erinaceus europaeus*, hedgehogs, betacoronaviruses, *Erinaceus* coronavirus, EriCoV, CD200 glycoprotein, CD200 ortholog, *Betacoronavirus* infection, *Eulipotyphla*

## Abstract

Due to their need for living cells, viruses have developed adaptive evolutionary strategies to survive and perpetuate in reservoir hosts that play a crucial role in the ecology of emerging pathogens. Pathogenic and potentially pandemic betacoronaviruses arose in humans in 2002 (SARS-CoV, disappeared in July 2003), 2012 (MERS-CoV, still circulating in Middle East areas), and 2019 (SARS-CoV-2, causing the current global pandemic). As universally recognized, bats host ancestors of the above-mentioned zoonotic viruses. However, hedgehogs have been recently identified in Europe and Asia as possible reservoirs of MERS-CoV-like strains classified as *Erinaceus* coronavirus (EriCoV). To elucidate the evolution and genetics of EriCoVs, NGS (next generation sequencing) and Sanger sequencing were used to examine fecal samples collected in Northern Italy in 2018/2019 from 12 hedgehogs previously found EriCoV-positive by RT-PCR. By sequence analysis, eight complete EriCoV genomes, obtained by NGS, showed a high phylogenetic correlation with EriCoV strains previously reported in Eurasia. Interestingly, eight viral strains presented an additional ORF encoding for the CD200 ortholog located between the genes encoding for the Spike and the ORF3a proteins. The CD200 ortholog sequences were closely similar to the host CD200 protein but varying among EriCoVs. The result, confirmed by Sanger sequencing, demonstrates for the first time that CoVs can acquire host genes potentially involved in the immune-modulatory cascade and possibly enabling the virus to escape the host defence.

## 1. Introduction

Coronaviruses (CoVs), classified as belonging to the Coronaviridae family, are a heterogeneous group of viruses with RNA (26–32 kb in length) single strand genomes which have been considered for a long time to be restricted to animals, in which that can cause respiratory, gastrointestinal, and other symptoms of variable severity [1]. In humans, CoVs are the second cause of respiratory disease causing about 15% of common colds worldwide [2]. Since 2002, three highly virulent strains classified into two subgenera (*Sarbecovirus* and *Merbecovirus*) of the *Betacoronavirus* (Beta-CoV) genus have caused severe respiratory diseases leading to thousands of infections and deaths worldwide. The *Sarbecovirus* subgenus includes the CoV species causing the severe acute respiratory syndrome (SARS-CoV) and the SARS-CoV-2, emerged in China in 2002 and in December 2019, respectively. The COVID-19 pandemic caused by SARS-CoV-2 is still ongoing. In 2012, the Middle East respiratory syndrome coronavirus (MERS-CoV), belonging to the *Merbecovirus* subgenus, was first reported in The Arabian Peninsula where it still circulates [3,4].

The ability of CoVs to change fast by recombination, deletion or by acquiring mutations enables the virus to jump among different species and successfully adapts to new hosts [5]. Since the SARS epidemic, it has become evident that several bat species host possible ancestors of these three zoonotic betacoronaviruses, mainly found in the Rinolophidae (SARS-CoV and SARS-CoV-2 precursors) and Vespertilionidae (MERS-CoV precursors) families of the order Chiroptera [6,7,8,9,10].

Hedgehogs are mammals belonging to the *Eulipotyphla* order which is phylogenetically related to the *Chiroptera* order [11] and have been recently identified as possible reservoir of MERS-CoV-like strains in Europe and Asia [12,13,14,15,16]. In particular, within the genus *Erinaceus* two species have been suggested as reservoirs of novel CoVs which have been clustered into a separate *Betacoronavirus* clade, among *Merbecovirus* members. The Western European hedgehogs’ CoVs were classified into the novel CoV species *Erinaceus* coronavirus (EriCoV) based on threshold value of 90% pairwise amino acid distance in the conserved domains of replicase polyprotein [17]. Up to date, the EriCoVs in hedgehogs have been detected in Germany, France, Great Britain, and Italy in *Erinaceus europaeus* [12,13,14] and a novel species named HKU31 has been reported in China in *Erinaceus amurensis* [15].

As previously stated, the ancient mammalian taxa of *Chiroptera* and *Eulipotyphla* show a close phylogenetic relationship [18,19]. Interestingly, common origin between bats and hedgehog merbecoviruses has also been hypothesized. In particular, the close similarity observed in the spike regions suggests that the bat viruses may have originated from a recombination event between CoV strains from the two animal hosts [15]. Bat and hedgehog species living in the temperate zone, such as the Italian ones [20,21,22], are mainly mammalian hibernators, sharing nocturnal habits and a mainly insectivorous diet. Furthermore, MERS-like CoVs have been previously detected in Italian bats [23].

After the first identification of EriCoV, several EriCoVs have been detected but only for five strains found in Germany, UK and China full genomes are available [12,14,15]. Thus, the availability of additional EriCoV full genome sequences could be important in elucidating the evolution and genetics of EriCoVs and the role of hedgehogs as reservoir and possible chronic shedding carrier of these potentially emerging RNA viruses.

For the above-mentioned reasons, the next generation sequencing approach was used to fully sequence genomes of betacoronaviruses detected in hedgehogs in Northern Italy in order to elucidate evolution, genetics of EriCoVs and to evaluate their role in public and veterinary health implications.

## 2. Materials and Methods

### 2.1. Samples

Between November 2018 and January 2019, 12 injured hedgehogs (*Erinaceus europaeus*) were found in both urban areas (cities, towns, villages, and suburbs) and rural areas in two provinces of the Emilia-Romagna region (Northern Italy). The hedgehogs were non-invasively sampled at a wildlife treatment and rehabilitation center and fecal samples were collected. Animals tested pan-CoV positive by RT-PCR [16] were enrolled in the study (Table 1). Total RNA was extracted by QIAmp Viral Mini kit (Qiagen, Milan, Italy) stored at −80 °C or immediately used.

### 2.2. Whole-Genome Sequencing

Libraries were prepared as previously described by Moreno et al. [23] following the sequence independent single primer amplification (SISPA) [24]. The RNA was reverse transcribed by SuperScript IV Reverse Transcriptase (Thermo Fisher Scientific, Monza, Italy) following the manufacturer’s instructions. The second strand of cDNA was synthesized by DNA Polymerase I Large (Klenow) Fragment (Promega, Milan, Italy) and the Klenow product amplified by the PCRBIO HiFi Polymerase (PCR BIOSYSTEM, Resnova s.r.l, Roma, Italy). The purified cDNA was digested with EcoRV enzyme (New England BioLabs, Pero, MI, Italy) and the libraries prepared by NEBNext Fast DNA Library Prep Set for Ion Torrent (New England BioLabs) size selecting the 400 nt amplicons. The emulsion PCR and the sequencing run were performed using the Ion 520 & Ion 530 Kit-OT2 according to the instructions (Ion Gene Studio S5 and Ion 530 Chip).

### 2.3. Complete Genome Assembling

Reads obtained by Ion Gene Studio S5 were analysed using the Galaxy Aries online tool (https://aries.iss.it/). The reads were checked, cleaned up and trimmed using the FastQC Read Quality reports and FASTQ positional and quality trimming. The de novo assembly was performed using the default parameters and excluding contigs shorter than 1000 bases by Spades 3.0. Reads were also mapped against the EriCoV reference genome (ErinaceusCoV/2012-174/GER/2012, NC_039207.1) using Bowtie2 software and the BAM output visualized by IGV software. The Open Reading Frames (ORFs) were predicted using the online tool ORF Finder (NCBI, http://www.ncbi.nlm.nih.gov/gorf/gorf.html). The complete genome Accession numbers on NCBI database were: ErinaceusCoV/Italy/116988-1/2018, MW246795; ErinaceusCoV/Italy/50265-19/2018, MW246796; ErinaceusCoV/Italy/50265-1/2018, MW246797; ErinaceusCoV/Italy/50265-17/2018, MW246798; ErinaceusCoV/Italy/50265-11/2019, MW246799; ErinaceusCoV/Italy/50265-12/2019, MW246800; ErinaceusCoV/Italy/50265-13/2019, MW246801; ErinaceusCoV/Italy/50265-15/2019, MW246802.

### 2.4. Recombination Analysis

Recombination analysis was performed with Simplot software version 3.5. by bootscan analysis. Complete genomes among *Merbecovirus* members were analysed with 1000 bootstrap replicates, a sliding window of 500 nucleotides and 200 nucleotides moving steps.

### 2.5. CD200 Detection by Reverse Transcription-PCR

Based on the alignment of the complete genome sequences of Italian EriCoVs, two primer pairs were designed to target the CD200 ortholog. The primer pairs were designed on the CoV genomes in the 5′ and 3′ flanking region of the CD200 gene allowing to amplify the 3′ end of the spike gene, the CD200 and the 5′ of ORF3a gene. The primer pairs were named CD200Fw (5′-TGGATGTGGCACTAGTTGTC-3′; position 25,507 on NC_039207.1; ErinaceusCoV/2012-174/GER/2012) and CD200Rw (5′-CTGGATATTAGGAGCTGTGT-3′; position 26000). The Reverse Transcription-PCR (RT-PCR) amplification was performed using the QIAGEN One-Step RT-PCR Kit (Qiagen) following the manufacturer’s instruction under the following amplification steps: 50 °C for 30′, 95 °C for 15′ and 40 cycles of 95 °C for 30″, 58 °C for 30″, 72 °C for 2′, and a final step of 72 °C for 7′. The amplicon obtained was 500 bp fragment if the CD200 ortholog was not present in the EriCoV genome. If the CD200 ortholog was present in the genome, and based on the insertion site, the expected size of the fragment will be >800 bp. The PCR products were analysed on 1% agarose gel. The DNA amplicons were sequenced using the same PCR primers used for the one step RT-PCR by Eurofins Genomic (Milano, Italy). The CD200 ortholog sequences Accession numbers on NCBI database were: ErinaceusCoV/Italy/50265-13/2019, MW294203; ErinaceusCoV/Italy/50265-12/2019, MW294204; ErinaceusCoV/Italy/50265-17/2018, MW294205; ErinaceusCoV/Italy/50265-4/2018, MW294206; ErinaceusCoV/Italy/50265-10/2018, MW294207; ErinaceusCoV/Italy/50265-1/2018, MW294208.

### 2.6. Phylogenetic Analysis

Sequence alignments, nucleotide/amino acid pairwise identities, and phylogenetic analysis (maximum likelihood) were performed using the MEGA7 software. Phylogenetic analyses were performed using the general time reversible model with a Gamma distribution and Invariant sites (GTR+G+I) with 1000 bootstrap replication as suggested by MEGA7 modeltest.

The most recent common ancestor (tMRCA) of *Merbecovirus* was estimated using the complete genome sequences and excluding the recombinant strains. The strict clock model with a constant-size coalescent tree prior was applied using the BEAST version 1.8.4 software (http://evolve. zoo.ox.ac.uk/beast/). The information about sampling dates were obtained from NCBI database (https://www.ncbi.nlm.nih.gov/nucleotide/) or published information. The generalized stepping-stone sampling (GSS) was used for model selection analysis and GTR + G + I as a substitution model. The Markov chain Monte Carlo (MCMC) was run with 100 million steps along and sampling every 10,000 steps. The convergence of MCMC chains was checked using Tracer v.1.7.1 checking ESS values >200 for each estimated parameter. The maximum clade credibility (MCC) tree was obtained from the tree posterior distribution using TreeAnnotator after 10% burn-in and tree displayed by FigTree software (http://tree.bio.ed.ac.uk/software/figtree/).

### 2.7. Structural Modelling

Analysis of the spike and CD200 proteins and the prediction of the secondary structure were performed using SWISS-MODEL (https://swissmodel.expasy.org) based on default parameters. EriCoV-RBD models were built with the MERS-RBD/human CD26 complex (4KR0). The structure and interacting residues of RBD analysis was performed in the models and analyzed and highlighted using Chimera software [25] Secondary structure elements are defined based on an ESPript (http://espript.ibcp.fr) algorithm [26] and compared to mouse CD200 (PDB: 4bfi).

## 3. Results

### 3.1. Complete Genome Sequences and Genome Organization

To determine the evolutionary relationship with other merbecoviruses, 12 fecal samples collected from Western European hedgehogs (*Erinaceus europaeus*) previously tested positive for CoVs [16] were subject to NGS to obtain complete genome sequences. From eight out of 12 samples, the sequence reads obtained enabled to build complete genomes (Table 1).

Sequence analysis of the full genomes by BLASTn showed the highest similarity to betacoronaviruses to the *Merbecovirus* subgenus. The complete genomes obtained are approximately 30 kb long with G + C content of approximately 37.7%. The genome organization of Italian hedgehog strains is similar to other members of the *Merbecovirus* subgenus: the 5′ UTR-ORF1ab-spike-ORF3a-ORF3b-ORF4a-ORF4b-ORF5-envelope (E)-membrane (M)-nucleocapsid (N)-ORF8b and the 3′ UTR. The ORF1ab gene has the predicted slippery sequence “UUUAAAC” involved in synthesis of the pp1ab polyprotein by ribosomal frameshift. A putative leader transcription regulatory sequence (TRS-L) 5′-AACGAAC-3′ preceded ORF1ab and seven putative transcription regulatory sequences body (TRS-B) have been identified for each ORF except for ORF4b and ORF3b, representing signals for the discontinuous transcription of subgenomic mRNAs (sgmRNAs) (Supplementary Table S1). Within the pp1ab protein, the size and genomic localization of the non-structural protein (NSP 1–16) were predicted with the expected cleavage sites of the “main protease” 3C–like protease (3CLpro) and the papain-like protease (Supplementary Table S2).

The Italian strains were collected from animals found in seven municipalities and genomes with almost identical nucleotide sequences from 95% to 97% identities (nt. id.) were obtained. Both identical (100%) and divergent (50265/1 and 50265/12; 96.1%) viral sequences were detected in each municipality. In four of them, only one strain was detected. Differently the same strain was detected (50265/1 and 50265/13 100% nt. id.) in animals captured in two different municipalities.

By sequence comparison, the viral sequences closest to the Italian sequence strains were from Western European hedgehog strains detected in German and Great Britain (MK6796601) displaying 92% and 91% nt. id. (NC_039207.1; ErinaceusCoV/2012-174/GER/2012), respectively, followed by 81% nt. id. with *Erinaceus amurensis* hedgehog coronavirus HKU31 (Ea-HedCoV HKU31) strain_F6 described in China (MK907286.1). The reference strains of MERS-CoVs (NC_019843.3, MERS_2c_EMC2012;) and the bat CoVs HKU4-1 and HKU5-1 (EF065505.1; EF065509.1) were more distant displaying 70% nt. id., 68% nt. id. and 67% nt. id., respectively. Similar results were obtained calculating the amino acids identities between the predicted protein of Italian strains and betacoronaviruses (Supplementary Table S3). The aa. id. values calculated comparing domains in the ORF1ab of the Italian strains with the hedgehog HKU31, MERS-CoV and MERS-CoV related sequence strains were lower than the threshold established for CoVs classification (90%), suggesting the classification in the EriCoV species represented by the prototype strain ErinaceusCoV/2012-174/GER/2012.

### 3.2. CD200 Ortholog Detection by RT-PCR

By reconstruction and analyses of the full genome sequences in six out of eight Italian strains (50265/1, 50265/11, 50265/12, 50265/13, 50265/17, 50265/19) an additional ORF was retrieved, placed between genes encoding for the Spike and the ORF3a protein preceded by TRS-B. The length of the novel ORF varied between 411 and 630 bp depending on the strain, encoding for a predicted peptide of 137-210 aa. By comparing the 6 sequences against the NCBI database the highest nucleotide identity ranged from 87.6% to 89.5% to the host *Erinaceus europaeus* CD200 gene and the highest aa. id. was of 74.1% to 80.3% to the translated host CD200 predicted protein (also named OX-2 membrane glycoprotein) (XP_007516410.2) (Supplementary Table S4). This result suggested the identification of the CD200 ortholog sequence in the genomes of the 6 fully sequenced EriCoVs.

To evaluate the presence of CD200 in the other 4 EriCoV strains for which the full genomes were not obtained, ad hoc primer pairs were designed onto the EriCoVs genome regions flanking the CD200 ortholog. The RT-PCR was designed to amplify a fragment >800 bp if the CD200 gene was inserted in the genome and a 500bp if it was not. By RT-PCR, two strains showed the amplification of 1200 bp fragment that as confirmed by sequencing using the Sanger method corresponds to the CD200 ortholog sequence gene and mapped in the same position of the genome (between the Spike and ORF3a genes) (Table 1). Differently, four strains did not include the CD200 ortholog, as confirmed by sequencing the 500 bp fragments obtained by RT-PCR (Table 1).

To confirm the presence of the CD200 on six strains sequenced by NGS, the same RT-PCR followed by Sanger sequencing was also performed on. The sequences obtained by both sequence approaches were identical (100% nt. id.) each other and the site of insertion of the CD200 between the genes encoding for the Spike and the ORF3a protein was also confirmed. The 8 CD200 ortholog sequences displayed from 78.2 to 100% nt. id. each other and 67.9% to 100% aa. id. (Supplementary Table S4). Only two sequences were identical. Furthermore, by amino acid alignment in one strain (50265/12) a deletion of 189 nucleotides in the CD200 was present. By clustering in the Maximum Likelihood phylogenetic tree built including the CD200 sequences reported in mammals and several viral orthologs, the evolutionary correlation between EriCoV CD200 orthologs and the *Erinaceus europaeus* CD200 was confirmed (Supplementary Figure S1).

Since no crystal of the CD200 protein from *Erinaceus* species is available, the predicted secondary structure of the mouse CD200 was used for the analyses. Comparison of the structures revealed that CD200 orthologs found in Italian EriCoVs showed a similar structure to the mouse CD200 including 9 beta strands (named A,B,C,C’,C”,D,E,F,G) in the first Ig-like domain of CD200, which is the domain involved in the binding of the host receptor CD200R. The 50265/12 strain predicted CD200 structure lacked the 8th and 9th (F, G) beta strands (Supplementary Figure S2). In addition, the amino acids stretch involved in the binding interface of complex CD200/CD200R are highly conserved between mouse CD200, hedgehogs and the CD200 orthologs of EriCoV (Table 2).

### 3.3. Phylogenetic Analysis of the Complete Genomes

**Figure 1:**
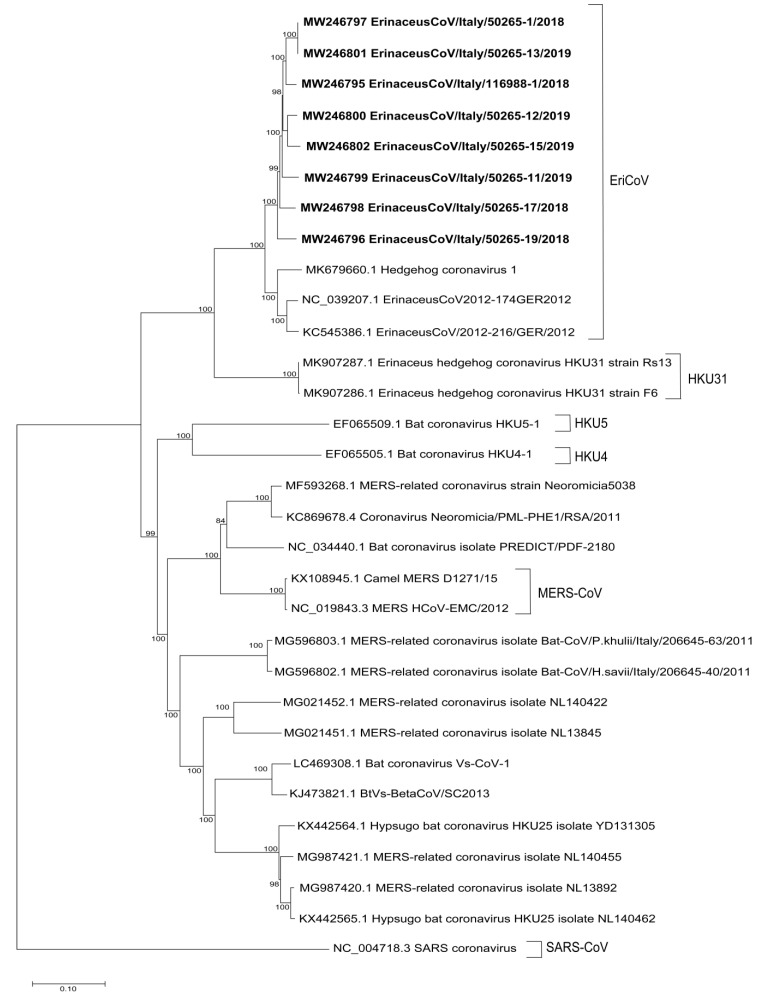
Maximum Likelihood phylogenetic tree of the *Merbecovirus* subgenus complete genomes including the eight Italian EriCoV strains. The tree was built using the GTR+G+I model and 1000 bootstrap replicates with supported nodes (>70%). The entries are reported as: Accession number and the sequence name.

Recombination events were excluded between Italian EriCoVs and either *Merbecovirus* or Italian bat strains (MG596803; Bat-CoV/P.khulii/Italy/206645-63/2011). The phylogenetic tree built using the complete genomes (Figure 1) showed the clustering of the Italian CoV hedgehog strains with the Hedgehog coronavirus 1 strain (MK679660.1), the ErinaceusCoV/2012-174/GER/2012 (NC_039207.1) and ErinaceusCoV/2012-216/GER/2012 (KC545386.1) while HKU31 and the other bat MERS-CoV related strains were not included in the cluster of EriCoVs.

The time of the most recent common ancestor (tMRCA) of *Merbecovirus*, separation of MERS and MERS related species from the EriCoV cluster was approximately 1447 years ago with highest posterior density (HPD) regions at 95% of 717 to 2390. The European EriCoVs originated approximately 190 years ago (95% HPD: 94 to 313) and the MERS-CoV and MERS-CoV related strains were separated from bat CoVs HKU4 and HKU5 1229 years ago (95% HPD: 610 to 2036) (Supplementary Figure S3).

### 3.4. Spike Protein Sequence Analysis

Similar results to the analyses with the 6 whole genomes were observed for the spike protein which shared the highest identity 92% aa. id. with *Erinaceus* CoV/2012-174/GER/2012, 91% aa. id. with Hedgehog coronavirus 1 strain and only 58% with MERS-CoVs. The same amino acid identity was obtained by analysing the RBD region.

The Italian EriCoVs RBD had four amino acids (Y491, P502, D525 and V540) out of 12 conserved residues needed to MERS-CoV for the binding to the human hDPP4 receptor (Table 3). However, comparing the structure of the EriCoVs with MERS-CoVs, the spike proteins lack the extended loop located between β6 and β7 needed for the interaction of hDPP4.

## 4. Discussion

Eight genomes of EriCoV strains were completely sequenced by NGS in order to evolutionarily characterize *Merbecovirus* members identified in Italian hedgehogs whose role as natural reservoirs and chronic carriers of betacoronavirus was previously hypothesized. Interestingly, genome analyses showed the common ORFs reported for other *Merbecovirus* strains and an additional ORF encoding the homologue of the CD200 of *Erinaceus europaeus* species, in six out of the eight completely sequenced genomes. The CD200 orthologs were also identified by conventional RT-PCR followed by Sanger sequencing in the other two CoVs that were unsuccessfully sequenced by NGS. Overall, the CD200 was identified by both sequencing the complete genomes (NGS) and the partial RT-PCR (Sanger sequencing) fragment in eight out of 12 EriCoVs tested. This finding is consistent with a template-switching mechanism deemed to underlie the high rate of RNA recombination in CoVs and consequently to represent a possible mechanism at the origin of recombinant CoVs including other CoV or even host related sequences [27,28].

The cellular CD200 protein, with an immune-modulatory effect, is able to suppress, by binding its receptor CD200R, the cascade of inflammatory responses and induce immune tolerance [29,30]. The CD200/CD200R interaction is an immunological checkpoint, which in the host prevents the excessive immune-mediated pathology induced as response of the host to infections. Indeed, the inhibitory effect of the CD200/CD200R may impair clearance of the virus. Many viruses have incorporated into their genomes the CD200 orthologs which have been shown to bind the host CD200R and cause downregulation of myeloid activity in the host [31,32,33,34] or to interact with the human CD200R diminishing the activation of macrophages and other immune cells [31,35,36,37]. Inhibition of the CD200-CD200R have a positive effect on coronavirus infections [38], restoring the IFN cascade and clearance of the virus, suggesting that it could also be a possible target for drugs which could reduce the damage caused by inflammatory response caused by the viral infection [39].

In the present study, analyses of the amino acid sequence and the predicted protein secondary structure suggest that the detected Italian EriCoV CD200 orthologs and the hedgehog CD200 could share the same beta strands and most of the sites involved in the binding interface with the mouse CD200R. Differently, one EriCoV strain detected (50265/1) showed a deletion of the beta strand involved in CD200R binding. However, the presence of an additional TSR-B sequence preceding each CD200 orthologs ORF is suggestive of an important role in the regulation of expression during the viral cycle.

**Figure 2:**
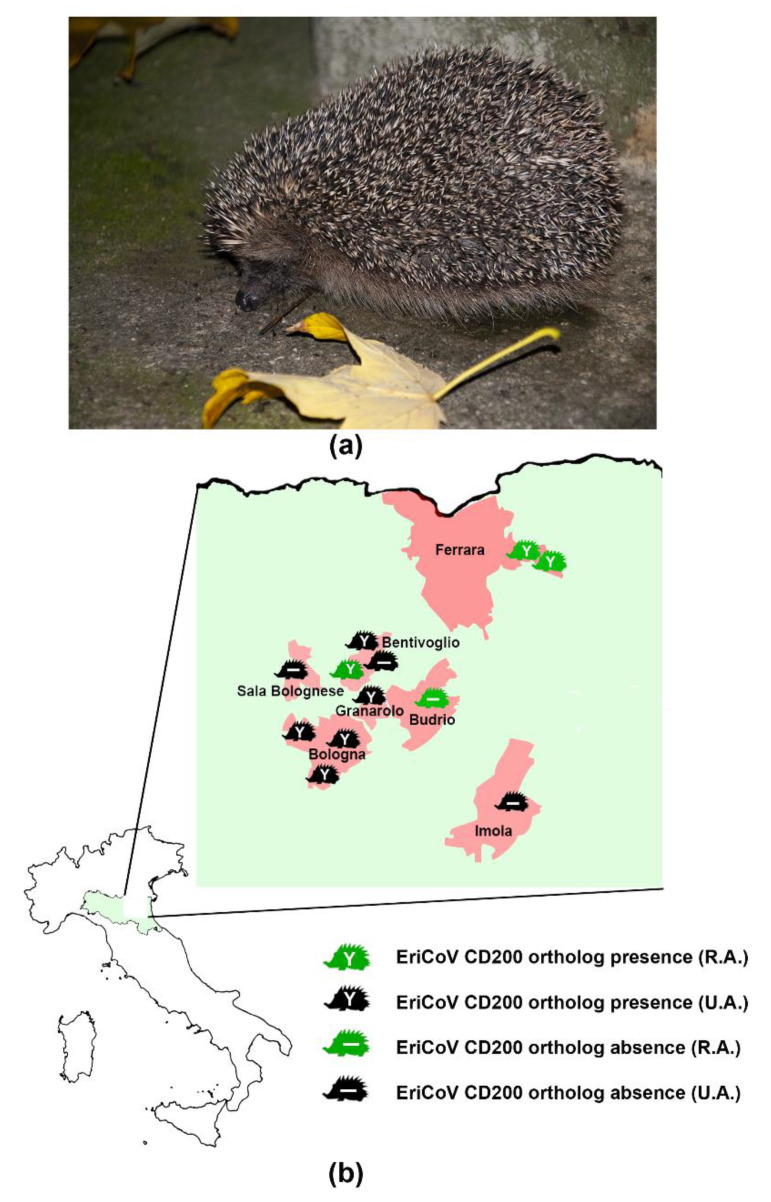
(**a**) Nocturnal image of Western European hedgehog (*Erinaceus europaeus*) from Northern Italy. (**b**) Viral CD200 ortholog in EriCoV strains from 12 hedgehogs sampled in rural areas (R.A.) and urban areas (U.A.) of Northern Italy. See Table 1 for details.

We hypothesize that the EriCoV CD200 may bind the host CD200R since the predicted encoded proteins of the CoVs have high amino acid identities and a highly similar secondary structure in the first Ig-like domain necessary for CD200R binding, whose prediction was based on the well characterized mouse CD200 [32]. To confirm this hypothesis in vitro studies are required [40,41]. It would be interesting to evaluate if this event, observed in hedgehogs from both urban and rural areas (Figure 2), is limited to EriCoV of this restricted study area or is widespread in hedgehogs’ CoV populations.

It is well known that CoVs as other RNA viruses are characterized by high evolutionary rate, due to the lack of proofreading activity of RNA-dependent RNA-polymerase. Although possible recombination events have been reported between bat CoVs and the CoVs from *E. amurensis* in China, in the present study recombination events between either Italian EriCoVs and MERS-related CoVs from Italian bats (Emilia Romagna region, 2011) [23] or other *Merbecovirus* strains were excluded.

Phylogenetic analysis conducted on the eight full genomes of the EriCoVs showed their evolutionary correlation with the other strains previously reported in German and Great Britain in *Erinaceus europaeus*. Differently, the EriCoVs detected are distant from the novel species CoV HKU31 that was only reported in Amur hedgehogs (*Erinaceus amurensis*) from China. This result, despite the few complete genomes available at the NCBI, may suggest that, as reported in bats, different hedgehogs’ species, occupying distant and unconnected areas, may be reservoir of different CoVs species.

By Bayesian analyses, the origin date of the EriCoVs was approximately 1829, confirming previous result [15] obtained with ORF1ab of HKU31 genomes, suggesting that the hedgehog CoVs may have recently emerged and suggesting that an even higher variability could arise in the future. More studies and sequences are required to understand the evolution and diversification of *Merbecovirus* members.

As resulted of sequence and phylogenetic analysis of the 8 Italian EriCoV strains obtained from animals captured in seven municipalities, different viral strains circulated in hedgehogs (95–97% nt. id.). Only two strains (50265/1, 50265/13) detected in animals captured in two municipalities (Bentivoglio, Granarolo) approximatively 9 km apart from each other were identical. The heterogeneity of EriCoV strains reported in our study, provide further evidences that hedgehogs are important wild reservoir of MERS-like CoVs. Further studies will be needed to better elucidate the heterogeneity of MERS-CoVs related strains in hedgehogs.

Finally, the possible implications for public and veterinary health related to the circulation of EriCoV in Italian hedgehogs were evaluated. We showed that prediction of the structure of the RBD domain, within the spike protein, of Italian EriCoVs presented a deletion of the loop region between β7 and β8 which is present in the MERS/HKU4-RDB and necessary for the receptor engagement [42]. The results lead to the hypothesis that hedgehog CoVs may not bind human receptor, however, since some CoVs may use co-receptors as well [43], this may not be a sufficient reason to prevent the virus from crossing the species barrier. Moreover, changing of the protein cannot be ruled out in the future and continuous surveillance is required to monitor the occurrence of mutations or other events, considering that few amino acid changes could determine the adaptation to novel hosts including humans. According to results previously obtained from a limited hedgehogs’ number, a sustained EriCoV circulation seems occur in the study area. Indeed, 58.3% of fecal samples collected in the Emilia-Romagna region of Northern Italy from 24 hedgehogs tested PCR-positive for EriCoVs, showing no clinical disease related to this infection [16]. These findings further support the hypothesis that hedgehogs could be considered natural reservoirs of CoVs and also act as chronic shedding carriers of these potentially emerging RNA viruses. In this context, particular attention must be paid to the management of hedgehogs admitted to multi-species wildlife rehabilitation centers [44], taking into account the high mutation rates characterizing members of the Coronaviridae family and their possible successful interspecies host jumps [45]. In particular, since hedgehogs and bats are primarily nocturnal and insectivorous mammals [20,21,22,46] playing the role of *Merbecovirus* reservoir, both direct and indirect contacts between them should be avoided to prevent possible cross species infection and recombination events. Finally, to avoid the man-made spread of EriCoVs into the wild, the return of rehabilitated hedgehogs to their original recovery areas is recommended.

## 5. Conclusions

We describe for the first time the acquisition of the host gene CD200 by Beta-CoVs. This event has been observed in a wild hedgehog population naturally infected with CoVs, sampled in Italy. The obtained result is important since evidenced the chance that CoVs can acquire and eventually use the host protein to their advantage. The incorporation of a gene involved with the immune-modulatory cascade could allow the virus to escape the host defence.

The acquisition by CoVs of a glycoprotein with multiple function types in the host needs further research, as it might affect the immunology, pathophysiology, and epidemiology of these potentially emerging pathogens. Further studies are required to better investigate the role played by these insectivorous mammals as CoVs’ reservoir and to delineate the animal origin of *Merbecovirus*.

Finally, taking into account the high mutation rates characterizing members of the *Coronaviridae* family and their possible successful interspecies host jumps, we have recommended precautionary health measures to be taken into account during the hedgehogs’ management at multi-species wildlife rehabilitation centers until their release into the wild.

## Figures and Tables

**Table 1:** Summary of NGS results and CD200 detection obtained by sequencing the 12 *Erinaceus* coronaviruses (EriCoVs) from hedgehogs sampled between November 2018 and January 2019. Sites of collection are also reported.

Sample ID	Municipality (Province) ^1^	Recovery Site ^2^	Total NGS Reads	N° CoVs Reads	% of Genome Built	CD200 Ortholog ^3^
116988/1	Budrio (BO)	R.A.	1,521,953	13,567	100%	-
50265/1	Bentivoglio (BO)	R.A.	1,246,304	13,094	100%	Y
50265/3	Sala Bolognese (BO)	U.A.	1,531,553	45	-	-
50265/4	Bologna (BO)	U.A.	1,679,321	320	-	Y
50265/10	Ferrara (FE)	R.A.	628,517	135	-	Y
50265/11	Ferrara (FE)	R.A.	1,506,517	174,733	100%	Y
50265/12	Bentivoglio (BO)	U.A.	979,563	78,706	100%	Y
50265/13	Granarolo (BO)	U.A.	1,635,986	16,679	100%	Y
50265/15	Imola (BO)	U.A.	1,046,044	85,354	100%	-
50265/16	Bentivoglio (BO)	U.A.	1,406,884	89	-	-
50265/17	Bologna (BO)	U.A.	1,677,168	136,279	100%	Y
50265/19	Bologna (BO)	U.A.	1,627,896	753,529	100%	Y

^1^ sites of animal findings; BO, Bologna; FE, Ferrara. ^2^ R.A., rural area; U.A., urban area. ^3^ Y present; - absent.

**Table 2:** Multiple sequence alignment showing variations in the key binding amino acid residues in CD200/CD200R binding site. Conserved residues are highlighted in red.

CD200														
Mouse	T33	Q35	N44	T47	N94	S41	P42	Q57	T95	G97	S98	Q99	K100	F96
50265/1	V52	Q54	N63	T66	N113	G60	P61	Q76	T114	G116	S117	G118	K119	F115
50265/4	T52	Q54	N63	T66	N112	I60	P61	Q76	T113	-	S115	I116	K120	Y114
50265/10	T52	Q54	N63	T66	N113	S60	T61	Q76	T114	G116	S117	G118	S119	F115
50265/11	T52	Q54	N63	T66	N113	G60	P61	Q76	I114	G116	P117	V118	M119	F115
50265/12	T52	T54	N63	T66	- ^1^	I60	P61	L76	-	-	-	-	-	-
50265/14	V52	Q54	N63	T66	N113	G60	P61	Q76	T114	G116	S117	G118	K119	F115
50265/17	T52	Q54	N63	T66	N113	V60	P61	Q76	T114	G116	S117	G118	R119	F115
50265/19	T52	Q54	N63	T66	N113	S60	P61	Q76	T114	-	S116	R117	K119	F115
Hedgehog	T60	Q62	N71	T74	N121	S68	P69	Q84	T122	G124	S125	G126	K127	F123

^1^ The dash marks a gap in the protein sequence.

**Table 3:** Multiple sequence alignment showing variations in key amino acid binding residues of the Spike protein. Conserved residues are highlighted in red.

MERS-CoV ^1^	Y 499	N 501	K 502	L 506	D 510	R 511	E 513	P 515	W 535	E 536	D 537	D 539	Y 540	R 542	W 553	V 555
Italian EriCoVs	Y 491	S 493	R 494	-	G 500	K 501	-	P 502	K 523	K 524	D 525	-	-	G 530	Y 538	V 540
HKU31 ^2^	Y 491	S 493	R 494	G 497	K 501	P 502	-	L 504	N 524	D 525	V 526	-	-	D 527	F 538	I 540
HKU4 ^3^	Y 503	S 505	K 506	L 510	N 514	Q 515	E 518	P 520	S 540	E 541	D 542	Q 544	V 545	K 547	L 558	I 560

^1^ Accession number: NC_019843.3, HCoV-EMC/2012. ^2^ Accession number: MK907286.1, HKU31 strain F6. ^3^ Accession number: EF065505.1, HKU4-1.

## Supplementary Materials

The following are available online at https://www.mdpi.com/1999-4915/12/12/1471/s1, Table S1: Genome localization of Italian EriCoVs predicted protein sequences, length and putative leader TRS, Table S2: Prediction of the putative pp1a/pp1b cleavage sites of Italian EriCoVs based on sequence comparison with Erinaceus/VMC/DEU/2012 (NC_039207.1), Table S3: Amino acids identities (%) between predicted proteins of the Italian EriCoVs and prototype clade c Betacoronaviruses, Table S4: Amino acids identities (%) between predicted CD200 ortholog from Italian EriCoVs and human, mouse and hedgehog CD200, Figure S1: Maximum Likelihood phylogenetic tree of CD200 sequences reported in mammals and viral orthologs, Figure S2: Alignment of amino acid sequences and secondary structure of CD200 orthologs found in Italian EriCoVs with the predicted beta strands (A, B, C, C’, C’’, D, E, F, G), Figure S3: Estimation of tMRCA of members of *Merbecovirus* using the complete genome sequences of Italian EriCoVs.
