# Supplementary material for: Can Coronaviruses Steal Genes from the Host as Evidenced in Western European Hedgehogs by EriCoV Genetic Characterization?
Source: Viruses. 2020 Dec 20;12(12):1471. doi: 10.3390/v12121471 (PMC7765933; doi:10.3390/v12121471)
Supplement: Supplementary file 1 [file viruses-12-01471-s001.pdf]

Supplementary Figures and Tables

Table S1: Genome localization of Italian EriCoVs predicted protein sequences, length and putative leader TRS

**Sample ID: 116988/1**			
**ORFs**	**Location on the genome**	**Length (aa)**	**TRS**
ORF1ab	221-21784	7188	41acgaa45
S	21704-25692	1329	21652atgaa21656
CD200 ortholog			
ORF3a	25803-26045	81	25698acgaa25702
ORF3b	25827-26240	138	
ORF4a	26004-26246	81	25995acgaa25999
ORF4b	26236-26904	223	
ORF5	26917-27596	226	26907acgaa26911
E	27671-27918	82	27661acgaa27665
M	27934-28589	218	27916acgaa27920
N	28645-29921	425	28622acgaa28626
ORF8b	28691-29251	187	

**Sample ID: 50265/1**			
**ORFs**	**Location on the genome**	**Length (aa)**	**TRS**
ORF1ab	220-21780	7187	40acgaa44
S	21700-25688	1329	21649atgaa21653
CD200 ortholog	25702-26303	200	25694atgaa25698
ORF3a	26307-26619	104	26294acgaa26298
ORF3b	26401-26821	140	
ORF4a	26578-26822	81	26569acgaa26573
ORF4b	26810-27479	223	
ORF5	27491-28170	226	27484acgaa27488
E	28245-28492	82	28238acgaa28242
M	28504-29159	218	28493acgaa28497
N	29214-30490	425	29194acgaa29198
ORF8b	29260-29821	187	

Sample ID: 50265/11			
**ORFs**	**Location on the genome**	**Length (aa)**	**TRS**
ORF1ab	221-21794	7191	41acgaa45
S	21710-25713	1334	21659acgaa21663
CD200 ortholog	25726-26318	197	25719aagaa25723
ORF3a	26322-26633	104	26309acgaa26313
ORF3b	26416-26836	140	
ORF4a	26593-26837	81	26583acgaa26587
ORF4b	26825-27493	223	
ORF5	27506-28183	226	27498acgaa27502
E	28260-28506	82	28252acgaa28256
M	28523-29175	218	28507acgaa28511
N	29233-30506	425	29212acgaa29216
ORF8b	29279-29838	186	

Sample ID: 50265/12			
**ORFs**	**Location on the genome**	**Length (aa)**	**TRS**
ORF1ab	229-21788	7187	49acgaa53
S	21709-25694	1328	21658atgaa21662
CD200 ortholog	25707-26116	137	25700aagaa25704
ORF3a	26123-26433	104	26110acgaa26114
ORF3b	26217-26637	140	
ORF4a	26394-26635	80	26385acgaa26389
ORF4b	26626-27298	223	
ORF5	27307-27988	226	27300acgaa27304
E	28061-28306	82	28054acgaa28058
M	28324-28976	218	28309acgaa28313
N	29034-30306	424	29014acgaa29018
ORF8b	29080-29639	186	

Sample ID: 50265/13			
**ORFs**	**Location on the genome**	**Length (aa)**	**TRS**
ORF1ab	220-21784	7189	40acgaa44
S	21706-25692	1329	21655atgaa21659
CD200 ortholog	25708-26308	200	25700acgaa25704
ORF3a	26313-26624	104	26300acgaa26304
ORF3b	26407-26824	140	
ORF4a	26584-26827	80	26575acgaa26579
ORF4b	26816-27483	222	
ORF5	26497-28178	227	27490acgaa27494
E	28251-28494	81	28244acgaa28248
M	28510-29161	217	28499acgaa28503
N	29220-30491	424	29200acgaa29204
ORF8b	29266-29826	187	

Sample ID: 50265/15			
**ORFs**	**Location on the genome**	**Length (aa)**	**TRS**
ORF1ab	224-21789	7189	44acgaa48
S	21707-25667	1320	21656acgaa21660
CD200 ortholog			
ORF3a	25773-26082	103	25760acgaa25764
ORF3b	25867-26284	139	
ORF4a	26044-26287	80	26035acgaa26039
ORF4b	26276-26942	222	
ORF5	26957-27635	226	26950acgaa26954
E	27711-27954	81	27703acgaa27707
M	27974-28629	218	27958acgaa27962
N	28684-29959	425	28663acgaa28667
ORF8b	28730-29330	200	

Sample ID: 50265/17			
**ORFs**	**Location on the genome**	**Length (aa)**	**TRS**
ORF1ab	229-21798	7190	49acgaa53
S	21715-25708	1331	21664atgaa21668
CD200 ortholog	25724-26354	210	25715atgaa25719
ORF3a	26369-26682	104	26356acgaa26360
ORF3b	26463-26882	140	
ORF4a	26640-26844	68	26631acgaa26635
ORF4b	26872-27547	225	
ORF5	27559-28242	228	27552acgaa27556
E	28319-28564	82	28312acgaa28316
M	28582-29237	218	28567acgaa28571
N	29292-30567	425	29272acgaa29276
ORF8b	29338-29900	188	

Sample ID: 50265/19			
**ORFs**	**Location on the genome**	**Length (aa)**	**TRS**
ORF1ab	224-21794	7190	44acgaa48
S	21710-25703	1331	21659atgaa21663
CD200 ortholog	25718-26314	199	25710acgaa25714
ORF3a	26330-26642	104	26317acgaa26321
ORF3b	26424-26844	140	
ORF4a	26601-26843	81	26592acgaa26596
ORF4b	26833-27505	224	
ORF5	27517-28205	229	27511acgaa27515
E	28280-28527	82	28274acgaa28278
M	28543-29198	218	28529acgaa28533
N	29253-30528	425	29234acgaa29238
ORF8b	29299-29861	187	

Table S2: Prediction of the putative pp1a/pp1b cleavage sites of Italian EriCoVs based on sequence comparison with Erinaceus/VMC/DEU/2012 (NC_039207.1)

NSP	Protein size (aa)	116988/1	50265/1	50265/11	50265/12	50265/13	50265/15	50265/17	50265/19	Putative functional domain(s)[a]
1	200	Met1 – Gly200	Met1 – Gly200	Met1 – Gly200	Met1 – Gly200	Met1 – Gly200	Met1 – Gly200	Met1 – Gly200	Met1 – Gly200	
2	659	Gly201-Gly859	Asp201-Gly859	Asp201-Gly859	Asp201-Gly859	Asp201-Gly859	Ala201-Gly858	Asp201-Gly859	Asp201-Gly859	
3	1983/1986	Ala860–Gly2843	Ala860–Gly2839	Ala860–Gly2844	Ala860–Gly2839	Ala860–Gly2839	Ala859–Gly2840	Ala860–Gly2843	Ala860–Gly2843	ADRP, PL2pro
4	504/506	Ser2844–Gln3310	Ser2840–Gln3303	Ser2845–Gln3310	Ser2840–Gln3303	Ser2840–Gln3310	Ser2841–Gln3304	Ser2844–Gln3307	Ser2844–Gln3310	
5	303/305	Ser3311–Gln3654	Ser3304–Gln3650	Ser3311–Gln3655	Ser3304–Gln3650	Ser3311–Gln3650	Ser3305–Gln3650	Ser3308–Gln3654	Ser3311–Gln3654	3CLpro
6	291	Ser3655–Gln3946	Ser3651–Gln3942	Ser3656–Gln3946	Ser3655–Gln3942	Ser3651–Gln3942	Ser3651–Gln3942	Ser3655–Gln3946	Ser3655–Gln3946	
7	82	Ser3938–Gln4029	Ser3934–Gln4025	Ser3938–Gln4029	Ser3934–Gln4025	Ser3934–Gln4025	Ser3934–Gln4025	Ser3938–Gln4029	Ser3938–Gln4029	
8	198	Ser4030–Gln4228	Ser4026–Gln4224	Ser4030–Gln4228	Ser4026–Gln4224	Ser4026–Gln4224	Ser4036–Gln4224	Ser4030–Gln4228	Ser4030–Gln4228	Primase
9	109	Asn4229–Gln4338	Asn4225–Gln4334	Asn4229–Gln4338	Asn4225–Gln4334	Asn4225–Gln4336	Asn4225–Gln4334	Asn4229–Gln4338	Asn4229–Gln4338	
10	139	Ala4339–His4478	Ala4335–His4474	Ala4339–His4478	Ala4335–His4474	Ala4337–His4476	Ala4335–His4474	Ala4339–His4478	Ala4339–His4478	
11	13	Ser4479–Leu4492	Ser4475–Leu4488	Ser4479–Leu4492	Ser4475–Leu4488	Ser4477–Leu4490	Ser4475–Leu4488	Ser4479–Leu4492	Ser4479–Leu4492	Short peptide at the end of ORF1a
12	933	Ser4493–Gln5412	Ser4489–Gln5408	Ser4493–Gln5412	Ser4489–Gln5408	Ser4491–Gln5410	Ser4489–Gln5408	Ser4493–Gln5412	Ser4493–Gln5412	RdRp
13	597	Ala5413–Gln6010	Ala5409–Gln6006	Ala5413–Gln6010	Ala5409–Gln6006	Ala5411–Gln6008	Ala5409–Gln6006	Ala5413–Gln6010	Ala5413–Gln6010	Hel, NTPase
14	523	Ser6011–Gln6534	Ser6007–Gln6530	Ser6011–Gln6534	Ser6007–Gln6530	Ser6009–Gln6532	Ser6007–Gln6530	Ser6011–Gln6534	Ser6011–Gln6534	ExoN, NMT
15	342	Gly6535–Gln6877	Gly6531–Gln6873	Gly6535–Gln6877	Gly6531–Gln6873	Gly6533–Gln6875	Gly6531–Gln6873	Gly6535–Gln6877	Gly6535–Gln6877	NendoU
16	310/311	Ala6878–Cys7188	Ala6874–Cys7183	Ala6878–Cys7189	Ala6874–Cys7183	Ala6876–Cys7185	Ala6874–Cys7185	Ala6878–Cys7189	Ala6878–Cys7189	OMT

[a]ADRP, ADP-ribose 1”-phosphatase; PL2pro, papain-like protease 2; 3CLpro, coronavirus nsp5 protease; Hel, helicase; NTPase, nucleoside triphosphatase; ExoN, exoribonuclease; NMT, N7 methyltransferase; NendoU, endoribonuclease; OMT, 2= *O*-methyltransferase.

Table S3: Amino acids identities (%) between predicted proteins of the Italian EriCoVs and prototype clade c Betacoronaviruses.

CoV strains[a]	ORFs	116988/1	50265/1	50265/11	50265/12	50265/13	50265/15	50265/17	50265/19
EriCoV	ORF1ab	95	94	94	94	94	94	94	95
	S	92	92	92	92	92	91	92	92
	ORF3a	84	91	90	87	91	90	89	90
	ORF3b	84	85	82	85	85	81	81	79
	ORF4a	84	83	83	86	86	83	82	79
	ORF4b	92	90	91	90	90	91	82	84
	ORF5	89	90	91	90	90	91	91	93
	E	96	96	96	96	96	95	96	96
	M	94	94	95	95	94	95	94	96
	N	94	94	96	95	94	94	94	94
	ORF8b	78	78	77	77	78	79	76	77
MERS-CoV	ORF1ab	75	75	75	75	75	75	75	75
	S	57	58	58	59	58	59	58	58
	ORF3a	34	33	32	31	33	32	33	31
	ORF3b	35	37	36	37	37	34	36	36
	ORF4a	37	40	39	40	40	38	39	40
	ORF4b	40	43	40	43	43	41	39	42
	ORF5	46	50	51	50	50	51	52	51
	E	74	74	74	74	74	73	74	74
	M	80	78	79	79	78	79	78	79
	N	74	72	72	72	72	72	73	72
	ORF8b	45	46	48	48	46	48	47	46
HKU31	ORF1ab	84	84	84	84	84	84	84	84
	S	80	80	81	80	80	81	80	81
	ORF3a	59	58	60	59	58	60	61	57
	ORF3b	69	71	67	71	71	67	71	67
	ORF4a	68	68	65	68	68	65	69	64
	ORF4b	73	73	72	73	73	73	72	74
	ORF5	75	76	77	76	76	76	77	77
	E	87	87	87	87	87	85	87	87
	M	94	94	94	94	94	93	94	92
	N	84	84	84	84	84	84	84	84
	ORF8b	59	58	60	59	58	58	59	61
HKU4	ORF1ab	72	70	70	70	70	70	70	70
	S	57	60	59	60	60	60	59	59
	ORF3a	33	25	26	25	25	26	26	23
	ORF3b	41	39	37	39	39	35	37	39
	ORF4a	37	44	43	44	44	40	43	43
	ORF4b	30	28	28	30	30	29	26	27
	ORF5	38	40	40	40	40	41	40	40
	E	61	62	62	62	62	61	62	62
	M	79	79	79	78	79	79	79	78
	N	70	70	71	70	70	70	70	71
	ORF8b	41	41	42	42	41	41	43	41
HKU5	ORF1ab	73	72	72	72	72	72	72	72
	S	58	59	59	59	59	59	58	59
	ORF3a	30	26	25	25	26	25	25	25
	ORF3b	47	39	37	39	39	36	36	37
	ORF4a	40	48	47	48	48	45	45	45
	ORF4b	28	28	27	28	28	28	26	28
	ORF5	45	48	48	48	48	48	47	47
	E	62	61	61	61	61	60	61	61

M	81	80	81	80	80	19	81	78
N	69	69	69	69	69	69	69	70
ORF8b	47	50	50	50	50	47	50	50

[a]Accession number of reference strains:

- EriCoV: NC_039207.1, Erinaceus/VMC/DEU/2012
- MERS-CoV: NC_019843, HCoV-EMC/2012
- HKU31: MK907286.1, HKU31 strain F6
- HKU4: EF065505.1, HKU4-1
- HKU5: EF065509.1, HKU5-1

Table S4: Amino acids identities (%) between predicted CD200 ortholog from Italian EriCoVs and human, mouse and hedgehog CD200.

CD200 host[a]	Homo sapiens	Mus Musculus	Erinaceus europaeus	50265/1	50265/4	50265/10	50265/11	50265/12	50265/17	50265/19
Mus Musculus	76.6									
Erinaceus europaeus	72.1	67.3								
50265/1	61.6	54.8	78.2							
50265/4	53.8	47.8	63.2	67.9						
50265/10	64.1	58.1	79.8	85.5	66.6					
50265/11	63.8	55.2	81.0	83.3	80.9	85.5				
50265/12	61.2	55.7	78.6	85.3	66.4	83.2	90.4			
50265/14	61.1	54.3	76.8	100	67.4	84.0	82.5	85.7		
50265/17	59.3	54.6	75.0	80.0	67.5	82.1	81.1	76.1	79.4	
50265/19	61.7	53.7	72.6	71.4	67.5	80.1	82.5	71.6	71.9	71.3

[a]Accession number:

- Homo sapiens: NP_005935, OX-2 membrane glycoprotein isoform
- Mus musculus: 4BFI, complex of the extracellular portions of mouse CD200R and mouse CD200
- Erinaceus_europaeus: XP_007516410, OX-2 membrane glycoprotein isoform X1

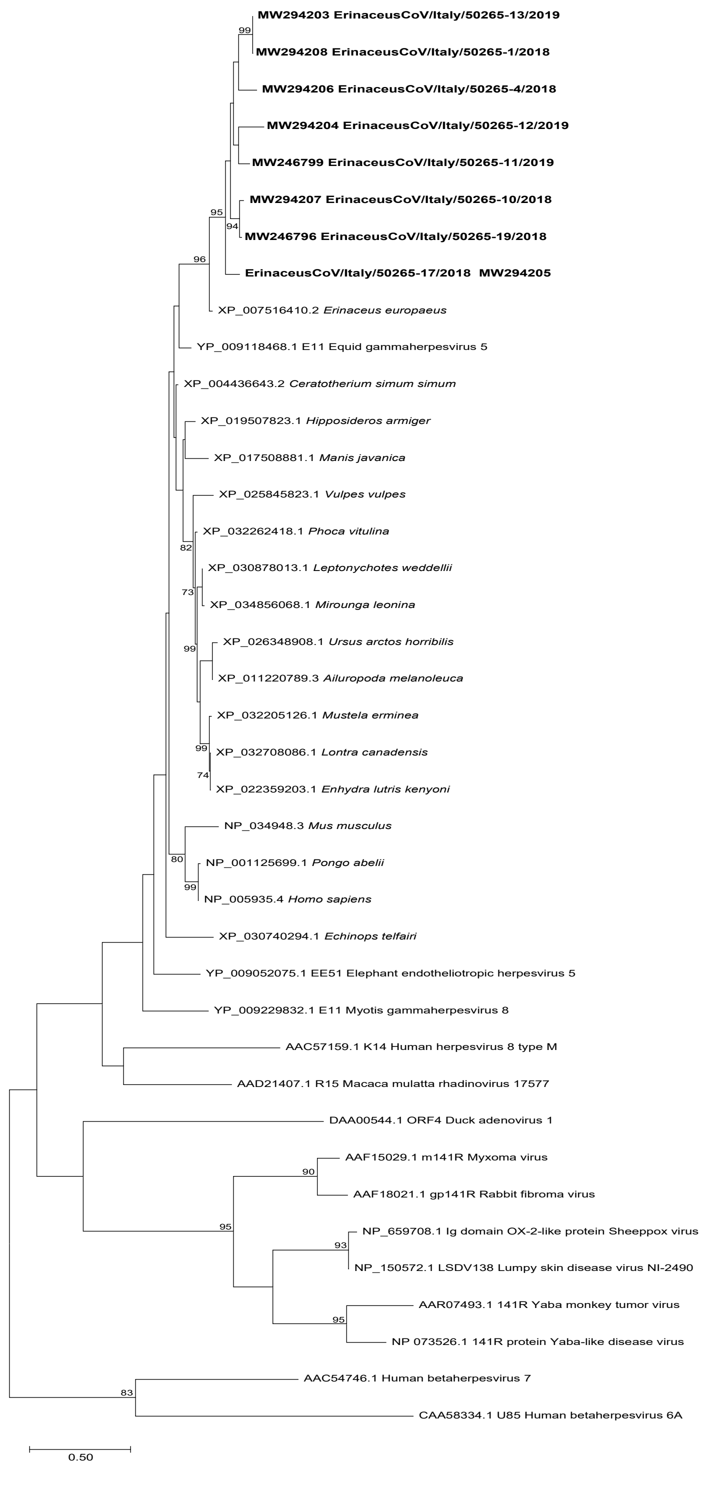

Figure S1: Maximum Likelihood phylogenetic tree of CD200 protein sequences reported in mammals and viral orthologs. The tree was built using the JTT+G model, as suggested by MEGA10 model test and 1000 bootstrap replicates with supported nodes (>70%). Sixteen CD200 sequences were included from mammals, 15 CD200 orthologs from viruses and the 8 CD200 reported in Italian EriCoVs from this study, reported in bold. The sequences are reported including the Accession number, mammal species or viral strain name.

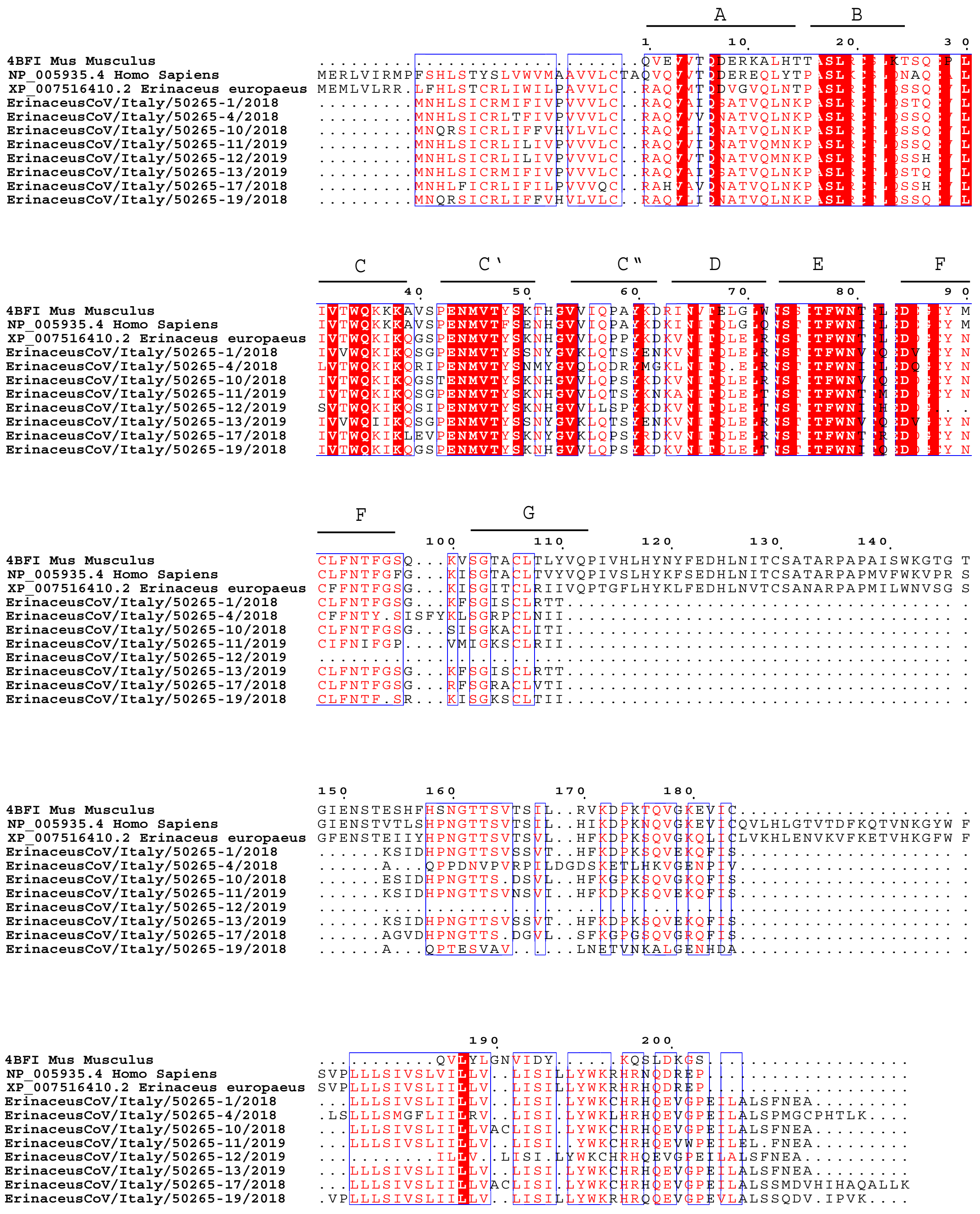

Figure S2: Alignment of amino acid sequences and secondary structure of CD200 orthologs found in Italian EriCoVs with the predicted beta strands (A, B, C, C', C'', D, E, F, G).

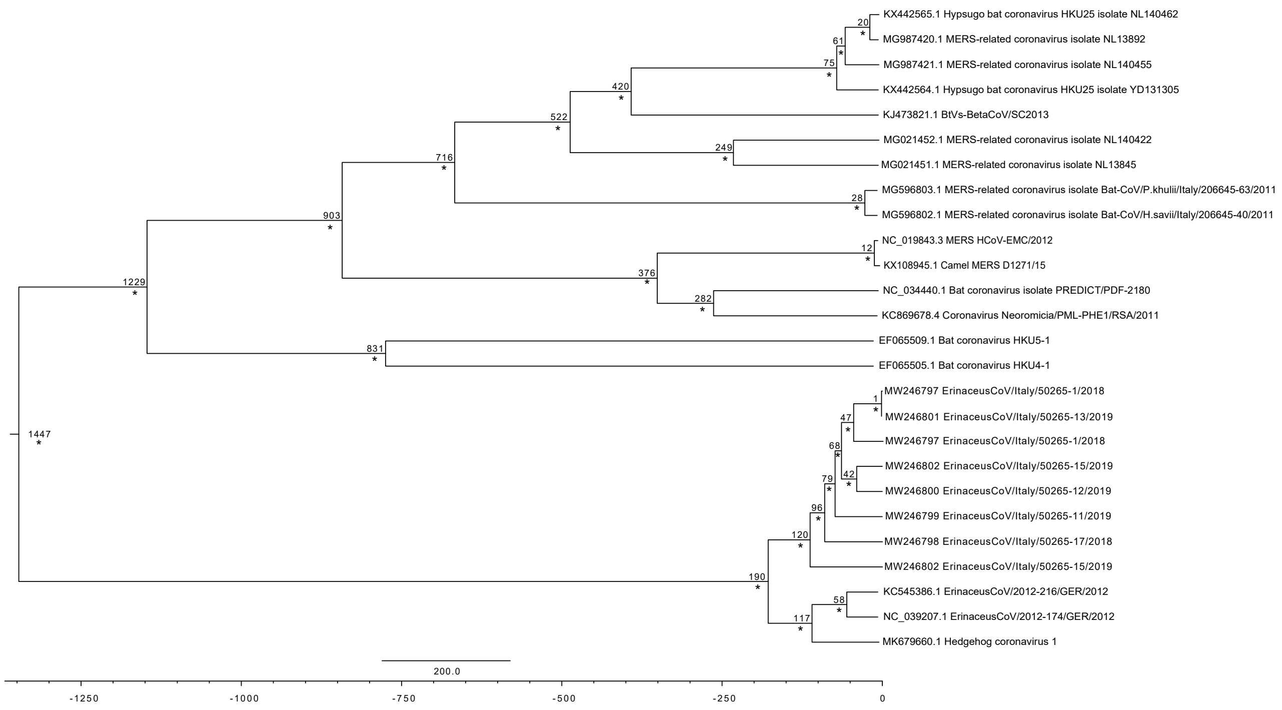

Figure S3: Estimation of tMRCA of members of *Merbecovirus* using the complete genome sequences including the Italian EriCoVs. The mean estimated dates were reported at the nodes. The asterisks indicate a posterior probability >0.9. The sequences are reported as Accession numbers, strain name and sampling dates.
